# Ocular surface inflammation induces de novo expression of substance P in the trigeminal primary afferents with large cell bodies

**DOI:** 10.1038/s41598-020-72295-x

**Published:** 2020-09-16

**Authors:** Yong-Soo Byun, Jee-Won Mok, So-Hyang Chung, Hyun-Seung Kim, Choun-Ki Joo

**Affiliations:** 1 Department of Ophthalmology and Visual Science, College of Medicine, Seoul St. Mary’s Hospital, The Catholic University of Korea, Banpo-daero 222, Seocho-gu, Seoul, 06591 Republic of Korea; 2 Catholic Institute of Visual Science, The Catholic University of Korea, Seoul, Republic of Korea; 3 CK St. Mary’s Eye Center, Seoul, Republic of Korea

**Keywords:** Corneal diseases, Animal disease models, Pain

## Abstract

We evaluated the changes in substance P (SP)-expressing trigeminal neurons (TNs) innervating the cornea following ocular surface inflammation. Ocular surface inflammation was induced in Sprague–Dawley rats using 0.1% benzalkonium chloride (BAK). The corneal staining score, corneal epithelial apoptosis, conjunctival goblet cells, and density of corneal subbasal nerve plexus (SNP) were assessed, and the mRNA levels of SP, interleukin (IL)-1β, IL-6, and tumour necrosis factor-α were measured in corneas and ipsilateral trigeminal ganglia (TG). SP-immunoreactivity (IR) was measured in corneal intraepithelial nerves and TNs. The cell size of corneal TNs in the TG was calculated. All parameters were observed immediately (BAK group), at 1 week (1 w group), and 2 months (2 m group) after 2 weeks of BAK application. BAK caused an increase in the corneal staining score and the number of apoptotic cells, loss of conjunctival goblet cells, reduced density of corneal SNP, and upregulated expression of SP and inflammatory cytokines in both the cornea and TG in the BAK group but those changes were not observed in the 2 m group. On the other hand, SP-IR% and mean cell size of corneal TNs increased significantly in the BAK, 1 w, and 2 m groups, compared to the control. Our data suggest that following ocular surface inflammation, large-sized corneal TNs which normally do not express SP, expressed it and this phenotype switching lasted even after the inflammation disappeared. Long-lasting phenotypic switch, as well as changes in the expression level of certain molecules should be addressed in future studies on the mechanism of corneal neuropathic pain.

## Introduction

Neuropathic pain is caused by the pathologies of the peripheral and central somatosensory nervous system^1^. Unlike nociceptive pain, which arises following the painful stimuli and exerts a protective role, neuropathic pain features abnormal hypersensitivity or pain from normally non-painful stimuli and accompanies chronicity^2^. Various maladaptive modifications, throughout the sensory pathway from peripheral sensory nerve to the brain, are involved during the process of changing normal pain response to neuropathic pain^3^. Aberrant regeneration following a peripheral axonal injury can lead to spontaneous activity and abnormal excitability, and the tissue damage accompanying a peripheral axonal injury can result in the release of various inflammatory mediators, such as cytokines, prostaglandins, substance P (SP), thereby, lowering the threshold potentials to stimuli^4–6^. Collectively, these changes can provoke alterations in axonal cell bodies, whereby silent receptors become activated and gene expression is altered resulting in peripheral sensitisation^7^. Lasting peripheral sensitisation, in turn, can lead to more complicated mechanisms in the central nervous system, referred to as central sensitisation^8^.

Recently, the underlying mechanism for neuropathic pain has been elucidated in the unexplained corneal pain that does not match the clinical sign^9,10^. Clinical studies using confocal microscopy have shown that aberrant changes in corneal nerves such as increased sub-basal nerve tortuosity, beading, branching, reflectivity, neuromas and nerve sprouting, and increase in inflammatory cells are frequently observed in patients who have chronic dry eye symptoms or persistent postsurgical pain with minimal or no ocular surface signs^11,12^. Corneal neuropathic pain has been identified in various conditions that lead to corneal nerve damage directly or indirectly via tissue inflammation, such as refractive surgery, dry eye disease, herpes virus, benzalkonium chloride (BAK) preserved eye drops, diabetes, and chemotherapy^9,13^.

The cornea is one of the most pain-sensitive tissues, densely innervated with peripheral axons of primary sensory neurons, having their cell bodies in the trigeminal ganglia (TG), and their central processes connected to the trigeminothalamic tract and the brain^14^. The peripheral endings of corneal nerves have heterogeneous nociceptors encoding different stimuli including temperature change, pressure, touch, and chemicals. Functionally, about 70% of primary sensory neurons innervating the cornea are polymodal nociceptors, which respond to multiple modalities of stimuli, mechanical energy, heat, chemical irritants, and chemical mediators^15,16^. Most of the polymodal nociceptor fibres belong to the unmyelinated C-fibre (80%), and some of those are thin myelinated Aδ-fibers^17^.

SP is an 11-amino acid neuropeptide that exerts various functions through interactions with neurokinin receptors^18^. SP is produced in a subset of polymodal nociceptors, the peptidergic nociceptors. It is synthesised in the cell bodies located in dorsal root ganglia (DRG) or TG and is then transported to central and peripheral processes via axonal transport mediating nociceptive transmission^19^. SP contributes to inflammatory or neuropathic pain through various mechanisms, and its antagonists are being investigated as potential drugs for relieving pain^20–32^.

SP has been identified in corneal nerve fibres and is associated with ocular inflammation, corneal wound healing, and corneal epithelial homeostasis^14,33^. Clinical studies have reported changes in tear SP in various ocular surface conditions, but an established relationship between SP levels and ocular symptoms or pain has not been yet determined^14^. Recent animal studies have shown that the changes in SP level are not only limited to the ocular surface but occur throughout the cornea-trigeminal sensory pathway following corneal injury and inflammation, thereby suggesting the connections between SP-expressing corneal sensory neurons and ocular pain^34–36^. Since SP can be produced in a variety of cells including neuronal cells, it is difficult to determine the profile of SP related to abnormal pain or neuropathic pain only with SP expression level^37^. Therefore, in addition to evaluating SP levels in the cornea and the ipsilateral TG, we aimed to evaluate whether the proportion and size distribution of primary sensory neurons expressing SP are altered following BAK-induced ocular surface inflammation. To the best of our knowledge, no study has yet shown the change in SP-expressing population of corneal trigeminal neurons following the ocular surface damage.

## Results

### Eye wiping behaviour

Six rats from each of the four groups (BAK, 1 w, 2 m groups, and control) were used for the eye wiping test, followed by an ocular surface assessment. The number of eye wipes for 30 s (mean ± standard deviation [SD]) was 8.2 ± 3.3, 15.5 ± 4.5, 12.2 ± 4.6, and 11.5 ± 3.6 in the control, BAK, 1w, and 2 m group, respectively. The difference was not significant among the groups (*P* = 0.0828).

### Ocular surface alteration

**Figure 1:**
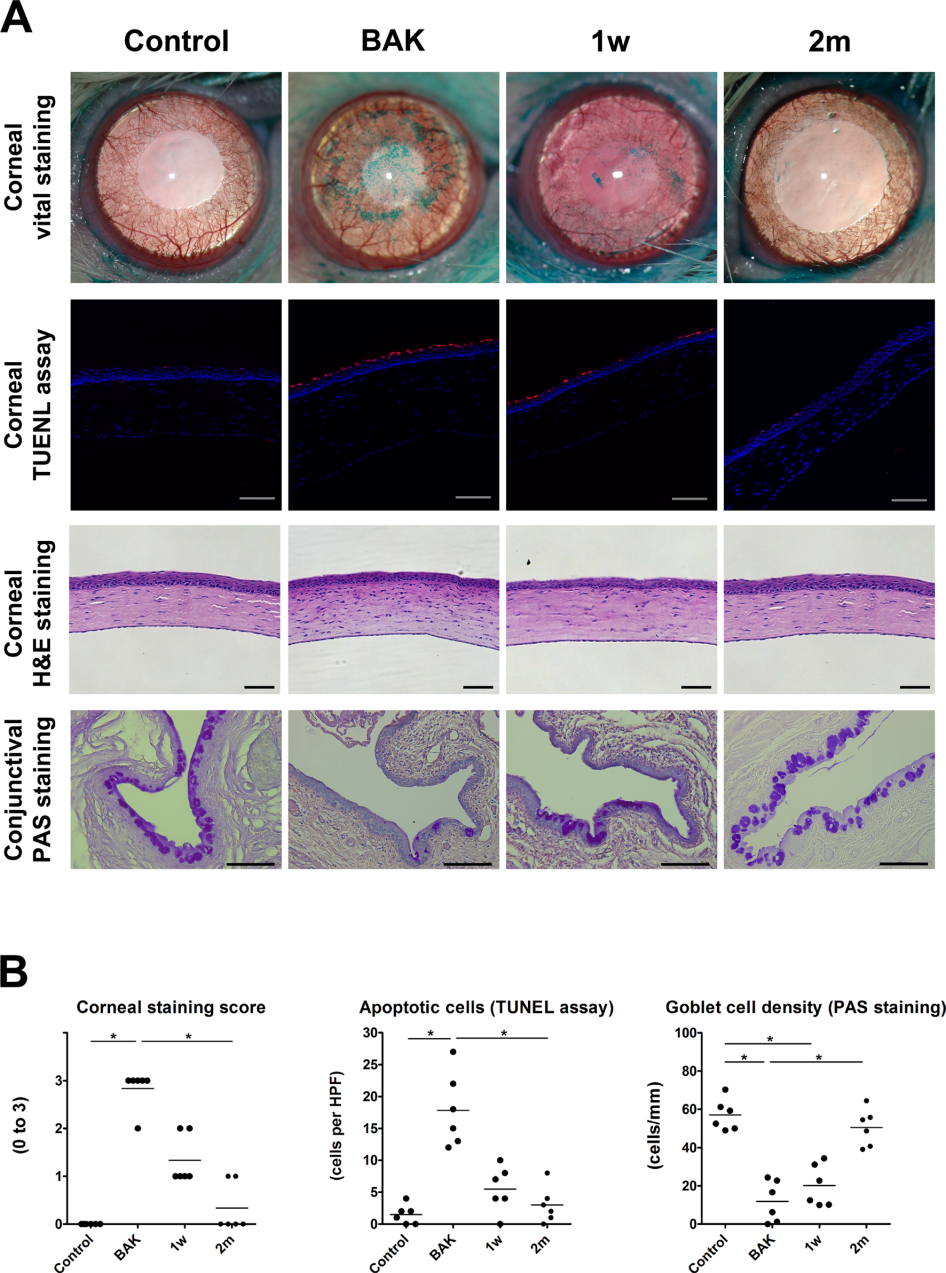
Benzalkonium-induced ocular surface changes. (**A**) The representative images of corneal vital staining, corneal TUNEL assay, corneal H&E staining, and conjunctival periodic acid-Schiff (PAS) staining in the control, BAK (benzalkonium), 1 w, and 2 m groups were taken by a camera-equipped surgical microscope (OPMI 1 FR pro, Carl Zeiss, Oberkochen, Germany), confocal laser scanning microscope (LSM800, Carl Zeiss), and an inverted light microscope (DMI 5000B, Leica, Wetzlar, Germany). In the BAK group, punctate epithelial erosions (green), apoptotic cells (red), and inflammatory cells with small round nucleus were markedly noted by vital staining, TUNEL assay, and H&E staining. Conjunctival goblet cells (violet) with PAS staining were greatly reduced in the BAK group. All scale bars = 100 μm. (**B**) The graphs of corneal staining score (left), the number of apoptotic cells (middle), and the goblet cell density (right) show that BAK treatment induced the ocular surface damage of the experimental rats and those alterations were not observed at 2 months after BAK treatment. *Significant difference between two groups by Dunn’s multiple comparison test as a post-hoc test of non-parametric ANOVA test (P < 0.05).

After the completion of the eye wiping test, the ocular surface was assessed according to the corneal staining score, TUNEL assay, haematoxylin & eosin (H&E) and PAS stain (n = 6 in each group). Compared to the control (0 ± 0), the corneal staining score was significantly higher in the BAK group (2.8 ± 0.4), but not in the 1 w (1.3 ± 0.5) and 2 m (0.3 ± 0.5) groups (Fig. 1). TUNEL assay showed that the number of apoptotic cells in the corneal epithelium (cells per high power field [HPF]) was significantly higher in the BAK group (17.8 ± 5.8) than in the control (1.5 ± 1.5), but the number of apoptotic cells was not different in the 1 w (5.5 ± 3.6) and 2 m (3.0 ± 2.8) groups. In addition, H&E staining showed that in the BAK group, many cells with small and round nucleus, thought to be inflammatory cells, were infiltrated in the anterior stroma, unlike keratocytes, which have a spindle-like nucleus and reside between aligned lamellae. Such cells were observed in fewer numbers in the 1 w group, and rarely in the 2 m and control groups. The density of conjunctival goblet cells (cells/mm), which was identified with periodic acid-Schiff (PAS) staining, was significantly lower in the BAK (11.9 ± 10.8) and 1 w (20.12 ± 10.9) groups than in the control (57.0 ± 8.2), but there was no difference between the 2 m group (50.5 ± 9.7) and the control group. The corneal staining score and number of apoptotic cells of corneal epithelium were significantly decreased in the 2 m group than in the BAK group, and the density of conjunctival goblet cells was significantly increased in the 2 m group than in the BAK group. Taken together, the BAK group showed significant deteriorations in the corneal epithelium and conjunctival goblet cells, whereas the 2 m group showed no significant difference in all those measurements compared to the control group.

### Density of corneal nerve and percentage of substance P-immunoreactivity

**Figure 2:**
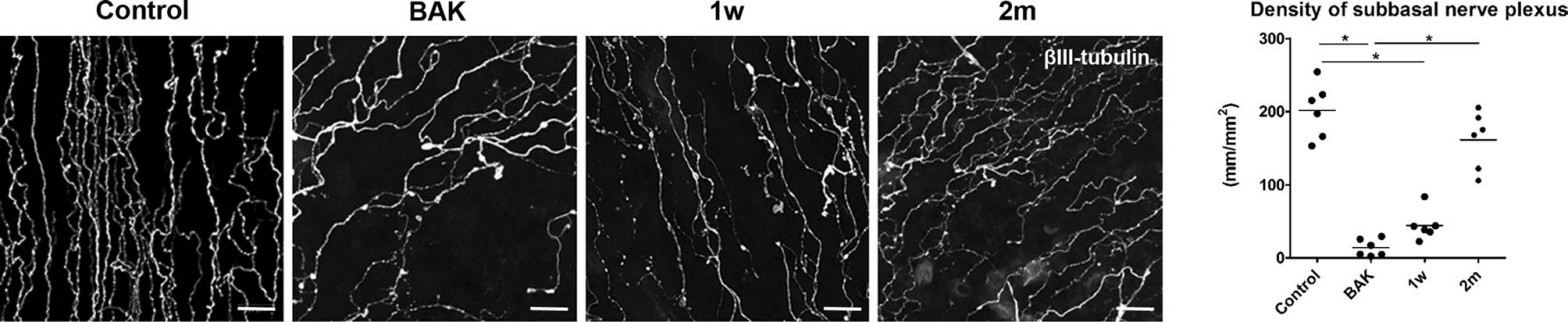
The density of corneal subbasal nerve plexus. The representative images of corneal subbasal nerve plexus (SNP) stained with βш-tubulin antibody in the control, BAK, 1 w, and 2 m groups. The density (length per area, mm/mm^2^) of corneal SNP decreased significantly after BAK treatment compared to the control, and at 2 months it was at near-control level. *Significant difference between paired groups by Dunn’s multiple comparison test as a post-hoc test of non-parametric ANOVA test (P < 0.05).

**Figure 3:**
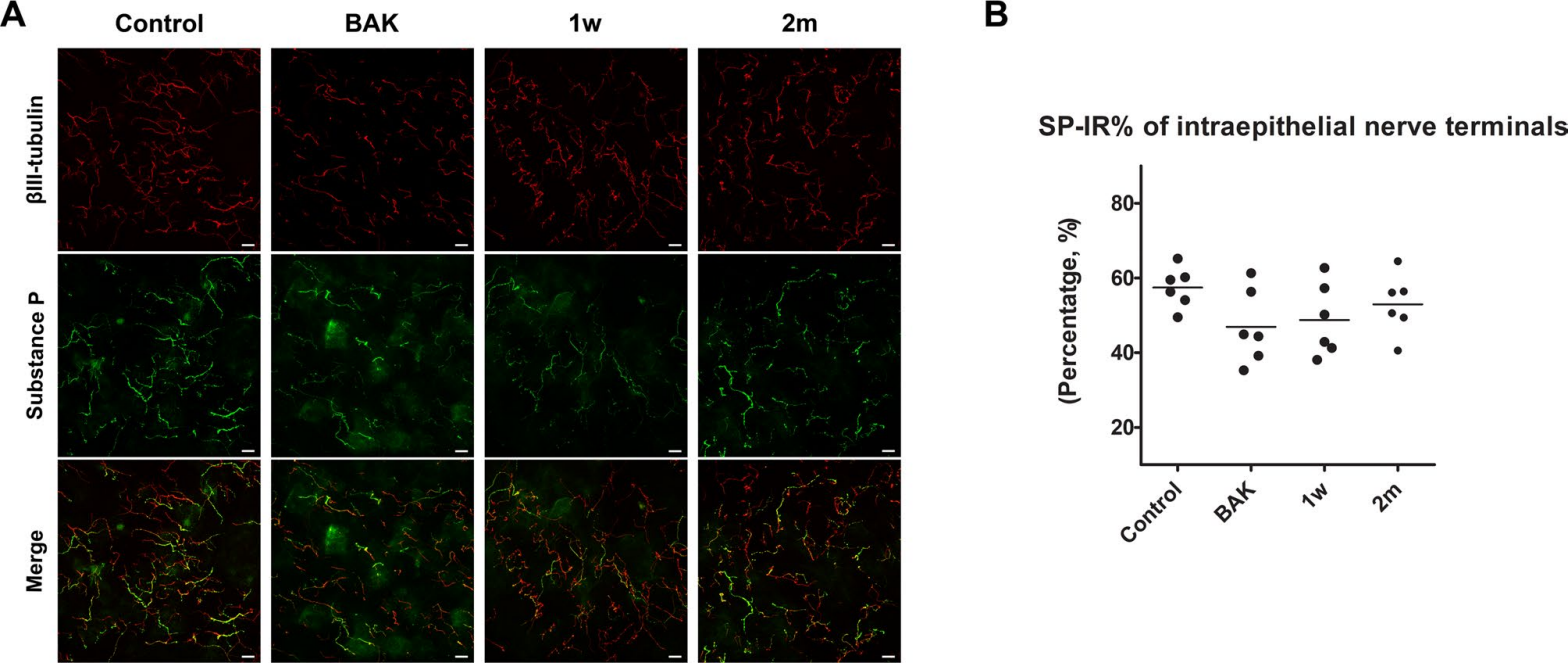
Immunolocalization of substance P in the corneal intraepithelial nerve terminals. The length of intraepithelial nerve terminals stained for substance P (SP) and βш-tubulin, a pan-neuronal marker, was measured and the SP-immunoreactivity (IR) (%) of corneal intraepithelial nerve terminals was calculated using Image J software. SP-IR (%) of corneal intraepithelial nerve terminals did not show any difference among the groups. In the BAK and 1 w groups, representing the acute phase of inflammation, SP-IR or background intensity was also observed in the neighbouring cells and extracellular matrix besides the nerve fibres. Scale bar = 20 μm.

The density and SP-immunoreactivity (IR) of corneal nerves were assessed in a different set of the four groups (n = 6). Figure 2 shows the representative images of the corneal subbasal nerve plexus (SNP) and its density in each group. The density of corneal SNP (mm/mm^2^) was significantly reduced in the BAK (13.9 ± 11.7) and 1 w (44.5 ± 20.8) groups, but not in the 2 m group (161.3 ± 39.09) compared to the control group (201.4 ± 37.7). The damage and recovery of corneal SNP corresponded to that of the ocular surface findings presented in Fig. 1. Most of the SNP contained SP-positive axons within their bundles; however, their fluorescence signal varied in intensity or appeared segmented. Therefore, we measured the SP-IR% of intraepithelial nerve terminals (Fig. 3), which was calculated as 46.9 ± 10.0, 48.8 ± 9.7, 52.9 ± 8.1, and 57.5 ± 5.4 in the BAK, 1 w, 2 m groups, and control, respectively. These values did not differ among all groups (P = 0.2543).

### Cell size distribution and substance P-immunoreactivity of corneal trigeminal neurons

**Figure 4:**
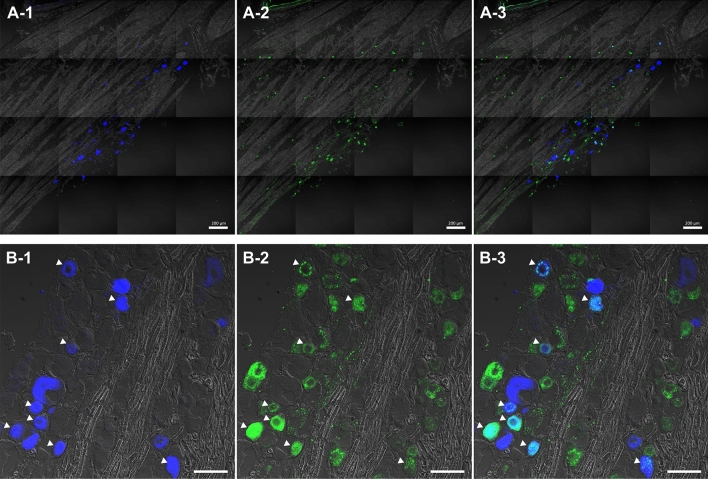
Fast-blue retrograde labelled cells and substance P-immunoreactivity in the trigeminal ganglion (**A-1**) Fast-blue (FB) dye was applied to the corneal surface, which transported retrogradely along the axonal fibres. FB-labelled cells in the trigeminal ganglion, which represent primary sensory neurons innervating the cornea, were localised in the ophthalmic division of trigeminal ganglion. (**A-2**) Substance P (SP, green) was identified throughout the trigeminal ganglion. (**B-1**, **B-2**) FB-labelled cells with SP (white arrows) and without SP were counted and their cross-sectional area was measured by referring to the merged phase-contrast image.

**Figure 5:**
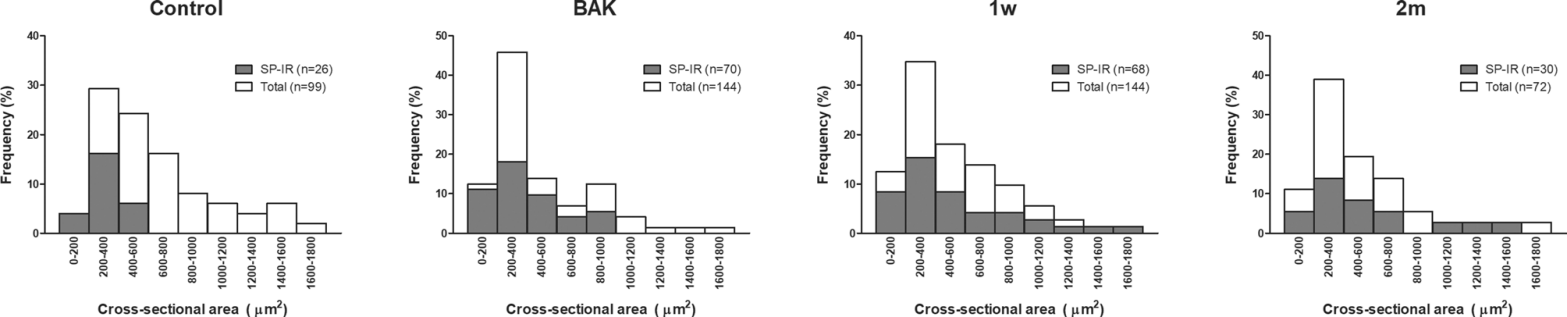
The histograms for the cell size of corneal trigeminal neurons with or without substance P-immunoreactivity after benzalkonium chloride-induced ocular surface inflammation. The frequency of fast blue (FB)-labelled trigeminal neurons with or without substance P-immunoreactivity (SP-IR) were sorted according to their cross-sectional area. Cumulative column, which represents primary sensory neurons innervating the cornea, showed the highest frequency in the range between 200 and 400 μm^2^, regardless of the groups. This pattern was also observed in FB-labelled neurons with SP-IR (solid column). The frequency of FB-labelled neurons with SP-IR above 800 μm^2^ increased in the BAK, 1 w, and 2 m groups (12.2%, 22.2%, and 21.4%, respectively) compared to the control group (0%).

Fast blue (FB)-labelled corneal trigeminal neurons were located in the ophthalmic division of TGs, and some population of these had SP-IR (Fig. 4). Table 1 shows the SP-IR% of FB-labelled neurons and cell size (mean ± SD, μm^2^) of FB-labelled neurons with or without SP-IR. Among FB-labelled cells, SP-IR% was significantly higher in all groups compared to control (P = 0.0005, 0.0012, and 0.0472 for the BAK, 1 w, and 2 m groups, respectively). Additionally, the cell size of FB-labelled cells with SP-IR was significantly greater in all groups compared to control (P = 0.049, 0.011, and 0.005 for the BAK, 1 w, and 2 m groups, respectively). In contrast, no difference was observed in the cell size of FB-labelled cells without SP-IR. Interestingly, analysis of the cell size distribution showed that the frequency of corneal trigeminal neurons with SP-IR above 800 μm^2^ increased in the BAK, 1 w, and 2 m groups (12.2%, 22.2%, and 21.4%, respectively) compared to the control group (0%), although corneal trigeminal neuron has the highest frequency in range 200 to 400 μm^2^, regardless of the groups (Fig. 5).

**Table 1:** Change in the proportion and cell size of fast blue-labelled trigeminal neuronal cells with or without substance P-immunoreactivity following benzalkonium chloride-induced ocular surface inflammation.

	Observed samples	Total number of FB-labelled cells	Number (%) of FB-labelled cells with SP-IR	Cell size of FB-labelled cells with SP-IR (μm^2^, mean ± SD)	Cell size of FB-labelled cells without SP-IR (μm^2^, mean ± SD)
Control	6	99	26 (26.3%)	309.1 ± 109.5	322.0 ± 89.1
BAK	6	144	70 (48.6%)*	415.6 ± 262.9^†^	327.2 ± 198.2
1 week	6	144	68 (47.2%)*	494.5 ± 343.8^†^	298.1 ± 137.1
2 months	6	72	30 (41.7%)*	518.7 ± 307.5^†^	311.8 ± 208.9

*BAK* benzalkonium chloride, *FB* fast blue, *SP-IR* substance P-immunoreactivity, *SD* standard deviation.

*Significant difference compared to the control group by Chi-square test (P < 0.05).

^†^Significant difference compared to the control group by Dunn’s multiple comparison test as a post-hoc test of non-parametric ANOVA test (P < 0.05).

### Expression of substance P and inflammatory cytokines in corneas and trigeminal ganglia

**Figure 6:**
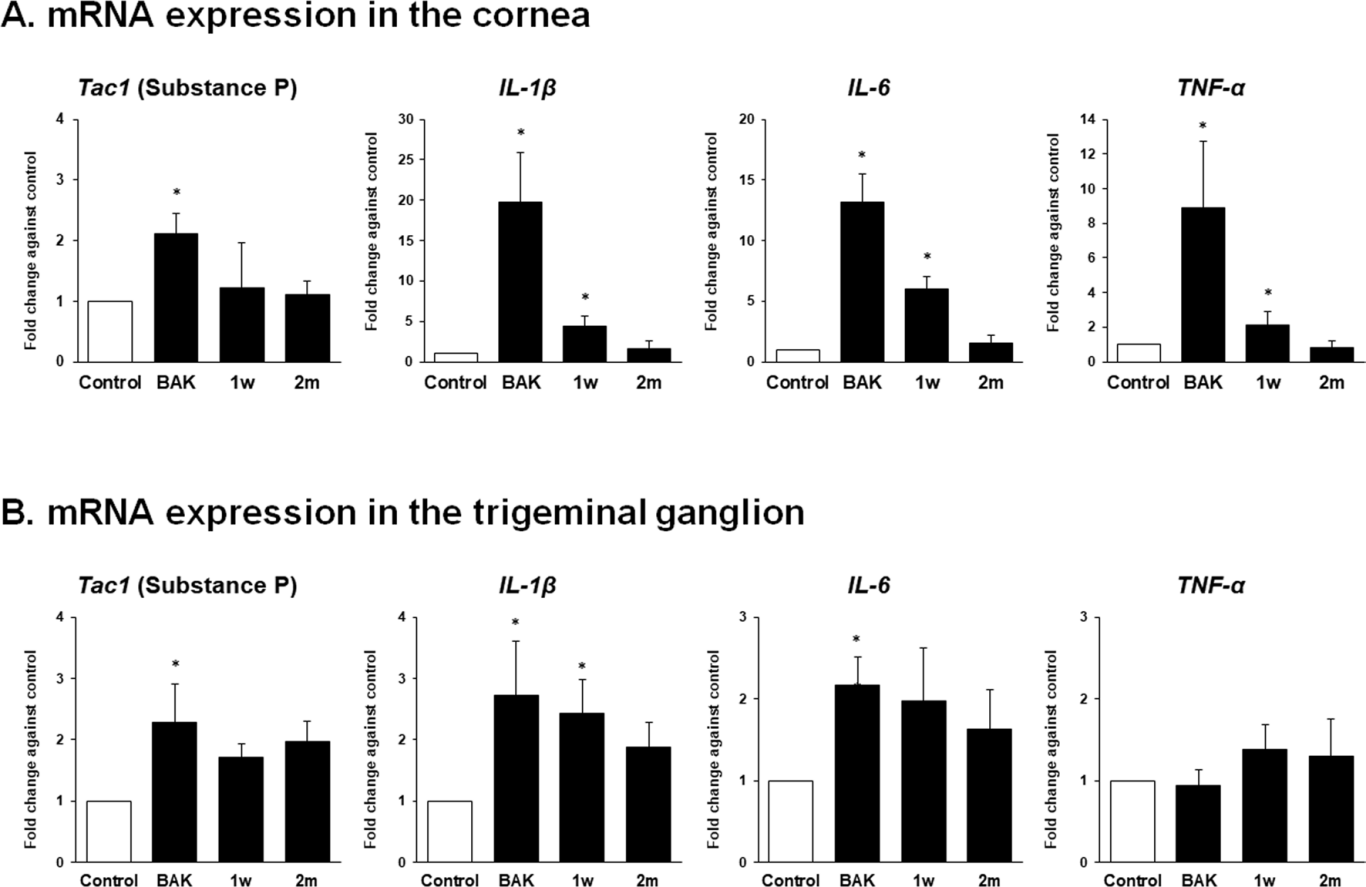
The mRNA expression level of substance P, IL-1β, IL-6, and TNF-α in the corneas and trigeminal ganglia after benzalkonium chloride-induced ocular surface inflammation. The fold change of *Tac1* (Substance P*), IL-1β*, *IL-6*, and *TNF-α* in the BAK, 1 w, and 2 m groups against the control group was calculated in the corneas (**A**) and the ipsilateral trigeminal ganglia (**B**). The values were derived from three tests, and three samples per test were used. *More than two-fold change vs. control.

The mRNA expression of SP and inflammatory cytokines (IL-1β, IL-6, and TNF-α) in corneas and ipsilateral TGs was determined using the formula ${2}^{{- {\Delta \Delta }C}_{T}}$ (Fig. 6). In corneas, SP (2.11 ± 0.34) showed > twofold upregulation in the BAK group, and no significant change was observed in the 1 w (1.22 ± 0.75) and 2 m (1.11 ± 0.22) groups. IL-1β, IL-6, and TNF-α showed > twofold upregulation in the BAK (19.74 ± 6.11, 13.21 ± 2.24, 8.87 ± 3.84, respectively) and 1 w groups (4.45 ± 1.15, 6.00 ± 1.00, 2.11 ± 0.81, respectively), whereas no significant fold change (1.68 ± 0.91, 1.53 ± 0.67, 0.79 ± 0.44, respectively) was observed in 2 m group, compared to control.

In the ipsilateral TG, SP showed > twofold upregulation in the BAK group compared to control, whereas no significant fold change in 1 w (1.72 ± 0.22) and 2 m (1.97 ± 0.34) groups was observed. IL-1β showed > twofold upregulation in the BAK (2.73 ± 0.88) and 1w (2.43 ± 0.54) groups and no significant fold change in the 2 m group (1.87 ± 0.41) compared to control. IL-6 showed > twofold upregulation in the BAK (2.17 ± 0.35) group, and no significant fold change in the 1w (1.98 ± 0.65) and 2 m groups (1.63 ± 0.48) compared to control. TNF-α showed no significant fold change among all groups (0.94 ± 0.20, 1.38 ± 0.31, and 1.30 ± 0.44 in the BAK, 1 w, 2 m group, respectively) compared to control.

## Discussion

This study demonstrated that the proportion and mean cell size of corneal trigeminal neurons with SP-IR were significantly increased in the BAK, 1 w, and 2 m groups, compared to the control. Unlike these changes in the SP-expressing corneal trigeminal neurons observed in all groups, the ocular surface deteriorations including corneal epithelial apoptosis, loss of conjunctival goblet cells, decreased density of intraepithelial corneal nerves, and increased expression of inflammatory cytokines were observed only in the BAK group, and not in the 2 m group. These findings suggest that BAK induced ocular surface inflammation/damage resulted in de novo SP expression in large-sized corneal trigeminal neurons, which normally do not express SP, and this change persisted even when ocular surface inflammation/damage was apparently resolved.

Normally, SP is present in about 25% of primary sensory neurons, and 0–32% of TG neurons depending on the species^38–42^. It has been reported that peripheral nerve injury or inflammation can induce de novo expression of SP in neuronal cells, which are normally negative for SP^43–45^. Bae et al.’s study of rat TG reported that 98% of neuronal cell bodies with SP-IR are small to medium-sized with the cross-sectional area < 800  μm^2^^38^. Our study showed that SP-IR neurons with the cross-sectional area > 800 μm^2^ were absent in control, but increased to 12.2%, 22.2%, and 21.4% in the BAK, 1 w, and 2 m groups, respectively. Therefore, it supports our assumption that the increase in proportion and mean cell size of SP-IR corneal trigeminal neurons indicates the phenotype switching of large neurons to the ones expressing it, although distinct phenotype of SP-expressing neurons was not characterised in our study. This is an important finding because the phenotype switching of a sensory neuron is known to contribute to nociceptive sensitisation, in which noxious stimuli generate greater response and even innocent stimuli elicit pain, and pain sensitivity can be extended to surrounding undamaged tissues^46^.

Unlike our speculation, the pattern of SP-IR% in intraepithelial nerve terminals was not consistent with that in TG. SP-IR% of intraepithelial nerve terminals was relatively constant among all groups. SP is synthesised in the perikaryon of primary afferent neurons in TG or DRG, then packed into storage vesicles, and axonally transported to both central and peripheral processes^47^. Owing to the process of its synthesis, transport, and release, the distribution of SP can vary along a neuron. Additionally, SP may be transported predominantly to the central process in large-sized cells with SP-IR. Neumann’s study^43^ showed that Aβ fibre, newly expressing SP after inflammation, could contribute to the excitability of spinal cord neurons, rather than exerting an efferent function at the periphery. Although these reports explain the inconsistency in the pattern of SP-IR% between the peripheral endings and cell bodies of corneal trigeminal neurons, studies on the expression pattern at the centre is required to elucidate it. Additionally, investigations of branching and tortuosity of corneal nerves, which have been widely accepted as important markers of peripheral neuropathy^48^ can be helpful in discovering peripheral changes corresponding to TG neuronal cell bodies.

Our results showed that inflammatory cytokines were slightly upregulated in the ipsilateral TG immediately and at 1 week after BAK treatment on the ocular surface, along with significant upregulation of inflammatory cytokines in the corneas. This result corresponds to previous studies, which have demonstrated that ocular inflammation/injury leads to the trigeminal and central inflammation along the cornea-trigeminal axis^36,49^. It has been widely accepted that SP can promote the production of inflammatory cytokines by activating immune cells, dependent or independent of their specific receptor, NK-1. In turn, inflammatory cytokines lead to SP production and release from sensory neurons and non-neuronal cells^50,51^. In this study, a slight increase in SP was observed in the BAK group along with an increase in the inflammatory cytokines in the BAK and 1 w groups, suggesting a possible link between inflammatory cytokines and SP expression. However, further studies into their sources and interaction will be needed to determine the connections between corneal inflammation and its expansion to the TG and neurochemical modification of neurons.

This study has some limitations. First, we were not able to determine whether changes in the SP expression profile of the corneal trigeminal neurons are related to an abnormal pain response. The eye wiping behaviour was used to indirectly assess pain sensitivity, but it did not show a significant difference between groups. The eye wipes appeared to be interrupted by other pain behaviours such as spasmodic blinking, prolonged eye closure, and burying heads in the sawdust. Therefore, standardised method assessing ocular pain or electrophysiological test should be included in future studies, as there is a recent advancement in developing experimental models for corneal pain studies^52^. Second, we did not evaluate the distinct subtype of large cells expressing SP due to limited resources. Recently, the latest analysis methods such as single-cell sequencing have further classified sensory neurons with the same neurochemical properties into distinct subtypes^53^. Although previous studies support our results making them interpretable, the distinct subtypes should be evaluated to expand our understanding of SP-related pain mechanism and approach the possible target for pain control.

In this study, we demonstrated that BAK-induced ocular surface inflammation caused SP expression in some population of large-sized TG neurons innervating the cornea, which normally do not express SP, and this phenotype switch lasted even after the ocular surface inflammation disappeared. This is a novel finding because to the best of our knowledge, no study has reported the long-lasting neuroplasticity related to ocular surface inflammation/damage. Clinically, many patients suffer from chronic dry eye symptoms or persistent postsurgical pain with minimal/no ocular surface signs. Although this phenotype switch of primary sensory neurons has already been addressed as a mechanism of pain pathology in other tissues, verification of it in the eye is an important step to expand our understanding of corneal nociceptive pain. Future studies on functional and neurochemical characterization of SP-expressing corneal neurons and inflammatory mechanisms that trigger their phenotypic change will provide insights into new therapeutic targets and approaches to corneal neuropathic pain.

## Methods

### Animals and experimental design

All procedures were performed in the semi-pathogen free zone of our animal care facility under the ARVO Statement for the Use of Animals in Ophthalmic and Vision Research. The study protocol was approved by the Institutional Animal Care and Use Committee (IACUC) of the College of Medicine, The Catholic University of Korea, Seoul, Republic of Korea (CUMC-2016-0314-01). BAK was used to induce toxic damage and nonspecific inflammation of ocular surface in Sprague–Dawley rats aged 6–8 weeks and weighing approximately 200 g. Unilateral eyes of rats were topically treated with 0.1% BAK ophthalmic solution (#63,249, Sigma-Aldrich, St. Louis, MO, USA) twice a day for 2 weeks under inhalation anaesthesia, isoflurane (JW Pharmaceutical Co., Seoul, Korea) according to a modified method of Xiong et al.^54^. Twenty-seven rats in each group were evaluated immediately (BAK group), at 1 week (1 w group), and 2 months (2 m group) after 2 weeks of BAK application. As the control group, 27 rats were assessed immediately after phosphate buffer saline (PBS) was applied to their unilateral eyes instead of BAK twice a day for 2 weeks. In each group, 6 eyes were used for the eye wiping test, corneal vital staining, TUNEL assay, HE and PAS staining; 6 for immunofluorescence staining of corneal nerves, 9 for RNA isolation, and 6 for immunofluorescence staining of TG with retrograde labelling.

### Eye wiping test

The eye wiping behaviour was observed to assess pain sensitivity according to Farazifard’s protocol^55^. Briefly, rat placed in a transparent cage was allowed to adapt to the experimental environment for 30 min, and then the number of eye wipes performed with forepaws was counted for 30 s after dropping 5 M NaCl solution into their eyes.

### Corneal vital staining

Following the eye wiping test, a vital dye, Lissamine green, (HUB Pharmaceuticals, LLC, Scottsdale, AZ, USA) was instilled into the inferior fornix after topical anaesthetic (0.5% proparacaine solution; Alcon, Geneva, Switzerland) was administered following the general anaesthesia, viz., intraperitoneal injection of 50 mg/kg tiletamine plus zolazepam (Virbac, Carros, France) and 15 mg/kg xylazine hydrochloride (Bayer, Leverkusen, Germany). Using images taken by a camera-equipped surgical microscope (OPMI 1 FR pro, Carl Zeiss, Oberkochen, Germany), corneal staining score was determined according to Van Bijsterveld grading system with modification^56,57^ as follows: absent: 0; sparsely scattered staining: 1; densely scattered, but not diffuse: 2; confluent or diffuse staining: 3; and a presence of plaques: 4. After corneal vital staining was completed, rats were euthanised by cervical dislocation and whole eyeballs were processed for TUNEL assay, H&E, and PAS staining.

### TUNEL assay and H&E and PAS stainings

Enucleated eyeballs (n = 6 per group) were immersed in fresh 2.5% glutaraldehyde solution overnight at 4 °C and were cut into 10 μm-thick paraffin sections from the vertical meridian of the eye. TUNEL assay was performed to discern the apoptotic cells in the cornea using the Click-iT TUNEL assay kit (C10246; Thermo-Fisher Scientific, Waltham, MA, USA), according to the manufacturer’s instructions. Images were captured using confocal laser scanning microscope (LSM800, Carl Zeiss), and TUNEL–positive cells (cells in an area of 600 × 600 μm^2^) in the corneal epithelium were counted. H&E and PAS staining (Sigma-Aldrich) were conducted to identify the presence of inflammatory cells in the cornea and the goblet cell density in the conjunctiva, respectively. Representative images were taken at the central cornea and the inferior conjunctival fornix in each group using a camera-equipped inverted light microscope (DMI 5000B, Leica, Wetzlar, Germany). The goblet cell density (cells/mm in an area of 400 × 300 μm^2^) was calculated as the number relative to the length of the conjunctival epithelial line using ZEN software (blue edition, Carl Zeiss) and Image J, ver. 1.47 (NIH, Bethesda, MA, USA).

### Immunofluorescence staining of corneal nerves

Corneas were processed for a flat mount, as described previously^58^. Briefly, corneas (n = 6 in each group) were trephined from the enucleated eyeballs and fixed in 4% paraformaldehyde (PFA) on ice for 1 h. After washing with PBS, the corneas were incubated in 0.1% EDTA (Sigma-Aldrich) and 0.02% hyaluronidase (type IV-S; Sigma-Aldrich) at 37 °C overnight, and further permeabilised in PBS with 0.3% Triton-X (PBST) containing 10% donkey serum and 1% bovine serum albumin (BSA; Jackson ImmunoResearch Laboratories, Inc., West Grove, PA, USA) for 2 h at RT. Further, corneas were incubated with primary antibodies in 0.1% PBST for 48 h at RT, followed by secondary antibodies in 0.1% PBST for 24 h at 4 °C. After incubation with antibodies, the corneas were washed extensively for 1 h in PBS, and then flattened by six radial cuts. Flat corneas were mounted on glass slides (Muto Pure Chemical Co., Ltd., Tokyo, Japan) using mounting media containing DAPI (Vector Laboratories, Burlingame, CA, USA), and images were captured using confocal laser scanning microscope. Mouse anti-β tubulin III, a pan-neuronal marker (1:200; ab78078, Abcam, Cambridge, UK) and rabbit anti-SP (1:400; ab67006, Abcam) were used as the primary antibodies. Fluorophore-conjugated secondary antibodies (1:400; Alexa Fluor 488 and 594, Abcam) were used according to the host species of the primary antibodies. Negative control staining was performed by omitting primary antibodies. Representative images (320 μm × 320 μm) were taken at the mid-periphery of each cornea using a confocal laser scanning microscope, and then processed with the z-axis projection to form the image corresponding to the corneal SNP and intraepithelial nerve terminals, following which, the density (total length/area, mm/mm^2^) of corneal SNP and SP-IR% (length/length) of intraepithelial nerve terminals were calculated using Image J.

### Immunofluorescence staining of trigeminal ganglia with retrograde labelling

Retrograde tracing method was used to label the TG neurons innervating the corneas (corneal trigeminal neurons) at 1 week before the immunofluorescence staining of TGs in each group (n = 6) according to the modified Gonzalez-Coto’s protocol^59^. Briefly, a 5 mm diameter-disc of Surgicel (Johnson & Johnson, New Brunswick, NJ, USA) soaked with 5% FB (FB; Polysciences, Inc., Warrington, PA, USA) was placed on the entire cornea for 2 h under general anaesthesia. After 1 week, which allowed FB to label the corneal trigeminal neurons retrogradely, TG was dissected following perfusion, fixation with 4% PFA, and processing for frozen sections. Ten-micrometer sections on the glass slides were washed thrice for 15 min and blocked in 0.1% PBST containing 10% donkey serum and 1% BSA for 30 min at RT. Then, the samples were incubated with anti-SP (1:400; Abcam) antibody in 0.1% PBST overnight at 4 °C, followed by secondary antibody in 0.1% PBST for 1 h at RT. After washing thrice for 5 min with PBS, samples on the glass slides were mounted in a medium without DAPI and examined using a confocal laser scanning microscope. The frequency of FB-labelled corneal trigeminal neurons with/without SP-IR and their cell size (the cross-sectional area, μm^2^) were measured using Image J.

### RNA isolation and quantitative real-time PCR

Nine cornea and ipsilateral TG each group were used to isolate RNA and analyse the mRNA level of SP and inflammatory cytokines using quantitative real-time PCR, according to the manufacturer’s instructions. Three corneas and TGs were used for a single test, and the result was averaged from three tests in each group. Tissue was homogenised in lysis buffer in a 1.5 mL tube. RNA extraction was done using RNeasy Mini Kit (#74,106, Qiagen, Hilden, Germany). The cDNA was synthesised from 1 μg total RNA using the RT2 First-strand cDNA synthesis kit (#330,401, Qiagen) and was mixed with primers and RT2 SYBR Green Mastermix (330,500, Qiagen). Each sample was assayed in duplicates using a real-time cycler (Bio-Rad, Hercules, CA, USA) under the conditions of 10 min at 95 °C, followed by 40 cycles of 95 °C for 15 s, and 60 °C for 1 min. The primers used were Tac1 for SP (PPR42621A, Qiagen), IL-1β (PPR06480B, Qiagen), IL-6 (PPR06483B, Qiagen), and TNF-α (PPR06411, Qiagen); and glyceraldehyde-3-phosphate dehydrogenase (GAPDH, PPR06557B, Qiagen) was used as the reference gene. The fold change with respect to control was calculated using the ${2}^{{- {\Delta \Delta }C}_{T}}$ method. More than twofold upregulation or less than 0.5-fold downregulation was considered as a significant change.

### Statistical analyses

All experimental data were analysed by non-parametric one-way analysis of variance (the *Kruskal–Wallis* test) after the normality of data was determined by D'Agostino & Pearson omnibus normality test. Pearson's Chi-squared test was used to compare the frequency among the groups. Two-sided P values < 0.05 were considered significant. All data are presented as mean ± SD. The data were analysed using the SPSS software (version 15.0, SPSS Inc., Chicago, IL, USA) and GraphPad Prism ver. 5.0 (GraphPad Software Inc., San Diego, CA, USA).

## Data Availability

The datasets used in the current study are available from the corresponding author on reasonable request.

## References

[1] Jensen TS (2011). A new definition of neuropathic pain. Pain.

[2] Campbell JN, Meyer RA (2006). Mechanisms of neuropathic pain. Neuron.

[3] Costigan M, Scholz J, Woolf CJ (2009). Neuropathic pain: a maladaptive response of the nervous system to damage. Annu. Rev. Neurosci..

[4] Opree A, Kress M (2000). Involvement of the proinflammatory cytokines tumor necrosis factor-alpha, IL-1 beta, and IL-6 but not IL-8 in the development of heat hyperalgesia: effects on heat-evoked calcitonin gene-related peptide release from rat skin. J. Neurosci..

[5] Lin CR (2006). Prostaglandin E2 receptor EP4 contributes to inflammatory pain hypersensitivity. J. Pharmacol. Exp. Ther..

[6] O'Connor TM (2004). The role of substance P in inflammatory disease. J. Cell Physiol..

[7] Truini A, Cruccu G (2006). Pathophysiological mechanisms of neuropathic pain. Neurol. Sci..

[8] Baron R, Hans G, Dickenson AH (2013). Peripheral input and its importance for central sensitization. Ann. Neurol..

[9] Rosenthal P, Borsook D (2016). Ocular neuropathic pain. Br. J. Ophthalmol..

[10] Goyal S, Hamrah P (2016). Understanding neuropathic corneal pain—gaps and current therapeutic approaches. Semin. Ophthalmol..

[11] Ross AR (2019). Clinical and in vivo confocal microscopic features of neuropathic corneal pain. Br. J. Ophthalmol..

[12] Chao C, Golebiowski B, Stapleton F (2014). The role of corneal innervation in LASIK-induced neuropathic dry eye. Ocul. Surf..

[13] Theophanous C, Jacobs DS, Hamrah P (2015). Corneal neuralgia after LASIK. Opt. Vis. Sci..

[14] Belmonte C (2017). TFOS DEWS II pain and sensation report. Ocul. Surf..

[15] Belmonte C, Aracil A, Acosta MC, Luna C, Gallar J (2004). Nerves and sensations from the eye surface. Ocul. Surf..

[16] Acosta MC, Tan ME, Belmonte C, Gallar J (2001). Sensations evoked by selective mechanical, chemical, and thermal stimulation of the conjunctiva and cornea. Invest. Ophthalmol. Vis. Sci..

[17] Belmonte C, Acosta MC, Gallar J (2004). Neural basis of sensation in intact and injured corneas. Exp. Eye Res..

[18] Hokfelt T (1982). Distribution of substance P in brain and periphery and its possible role as a co-transmitter. Ciba Found. Symp..

[19] Seybold VS (2009). The role of peptides in central sensitization. Handb. Exp. Pharmacol..

[20] Cao YQ (1998). Primary afferent tachykinins are required to experience moderate to intense pain. Nature.

[21] Li WW (2015). Substance P spinal signaling induces glial activation and nociceptive sensitization after fracture. Neuroscience.

[22] Li WW (2012). Substance P signaling controls mast cell activation, degranulation, and nociceptive sensitization in a rat fracture model of complex regional pain syndrome. Anesthesiology.

[23] Caudle RM (2010). Central sensitization in the trigeminal nucleus caudalis produced by a conjugate of substance P and the A subunit of cholera toxin. J. Pain..

[24] Sahbaie P (2009). Role of substance P signaling in enhanced nociceptive sensitization and local cytokine production after incision. Pain.

[25] Khasabov SG (2002). Spinal neurons that possess the substance P receptor are required for the development of central sensitization. J. Neurosci..

[26] Cohen RH, Perl ER (1990). Contributions of arachidonic acid derivatives and substance P to the sensitization of cutaneous nociceptors. J. Neurophysiol..

[27] Park CK (2010). Substance P sensitizes P2X3 in nociceptive trigeminal neurons. J. Dent. Res..

[28] Nakamura-Craig M, Smith TW (1989). Substance P and peripheral inflammatory hyperalgesia. Pain.

[29] Nakamura Y (2013). Volume transmission of substance P in striatum induced by intraplantar formalin injection attenuates nociceptive responses via activation of the neurokinin 1 receptor. J. Pharmacol. Sci..

[30] King AE, Ackley MA, Slack JR (1997). Profile of neuronal excitation following selective activation of the neurokinin-1 receptor in rat deep dorsal horn in vitro. Brain Res..

[31] Noguchi K, Kawai Y, Fukuoka T, Senba E, Miki K (1995). Substance P induced by peripheral nerve injury in primary afferent sensory neurons and its effect on dorsal column nucleus neurons. J. Neurosci..

[32] Pawlak M, Schmidt RF, Heppelmann B, Hanesch U (2001). The neurokinin-1 receptor antagonist RP 67580 reduces the sensitization of primary afferents by substance P in the rat. Eur. J. Pain..

[33] Suvas S (2017). Role of substance P neuropeptide in inflammation, wound healing, and tissue homeostasis. J. Immunol..

[34] He J, Pham TL, Kakazu AH, Bazan HEP (2019). Remodeling of substance P sensory nerves and transient receptor potential melastatin 8 (TRPM8) cold receptors after corneal experimental surgery. Invest. Opthalmol. Vis. Sci..

[35] Fakih D (2019). Chronic dry eye induced corneal hypersensitivity, neuroinflammatory responses, and synaptic plasticity in the mouse trigeminal brainstem. J. Neuroinflamm..

[36] Launay P-S (2016). Ocular inflammation induces trigeminal pain, peripheral and central neuroinflammatory mechanisms. Neurobiol. Dis..

[37] Zieglgansberger W (2019). Substance P and pain chronicity. Cell Tissue Res..

[38] Bae JY, Kim JH, Cho YS, Mah W, Bae YC (2015). Quantitative analysis of afferents expressing substance P, calcitonin gene-related peptide, isolectin B4, neurofilament 200, and Peripherin in the sensory root of the rat trigeminal ganglion. J. Comp. Neurol..

[39] Ng YK, Wong WC, Ling EA (1993). A qualitative and quantitative study of substance P immuno-cytochemistry of the trigeminal ganglion in the monkey. Anat. Embryol..

[40] Tervo T (1983). Substance P immunoreaction and acetylcholinesterase activity in the cornea and Gasserian ganglion. Ophthal. Res..

[41] Lehtosalo JI (1984). Substance P-like immunoreactive trigeminal ganglion cells supplying the cornea. Histochemistry.

[42] Felipe C, Gonzalez GG, Gallar J, Belmonte C (1999). Quantification and immunocytochemical characteristics of trigeminal ganglion neurons projecting to the cornea: effect of corneal wounding. Eur. J. Pain..

[43] Neumann S, Doubell TP, Leslie T, Woolf CJ (1996). Inflammatory pain hypersensitivity mediated by phenotypic switch in myelinated primary sensory neurons. Nature.

[44] Xu G-Y, Huang L-YM, Zhao Z-Q (2000). Activation of silent mechanoreceptive cat C and Aδ sensory neurons and their substance P expression following peripheral inflammation. J. Physiol..

[45] Ruscheweyh R, Forsthuber L, Schoffnegger D, Sandkuhler J (2007). Modification of classical neurochemical markers in identified primary afferent neurons with Abeta-, Adelta-, and C-fibers after chronic constriction injury in mice. J. Comp. Neurol..

[46] Weisshaar CL, Winkelstein BA (2014). Ablating spinal NK1-bearing neurons eliminates the development of pain and reduces spinal neuronal hyperexcitability and inflammation from mechanical joint injury in the rat. J. Pain..

[47] Harrison SS (2001). Substance P. Int. J. Biochem. Cell Biol..

[48] Hamrah P (2010). Corneal sensation and subbasal nerve alterations in patients with herpes simplex keratitis: an in vivo confocal microscopy study. Ophthalmology.

[49] Ferrari G (2014). Ocular surface injury induces inflammation in the brain: in vivo and ex vivo evidence of a corneal-trigeminal axis. Invest. Ophthalmol. Vis. Sci..

[50] Green DP, Limjunyawong N, Gour N, Pundir P, Dong X (2019). A mast-cell-specific receptor mediates neurogenic inflammation and pain. Neuron.

[51] Fiebich BL, Schleicher S, Butcher RD, Craig A, Lieb K (2000). The neuropeptide substance P activates p38 mitogen-activated protein kinase resulting in IL-6 expression independently from NF-kappa B. J. Immunol..

[52] McKay TB (2019). Corneal pain and experimental model development. Prog. Retin. Eye Res..

[53] Usoskin D (2015). Unbiased classification of sensory neuron types by large-scale single-cell RNA sequencing. Nat. Neurosci..

[54] Xiong C (2008). A rabbit dry eye model induced by topical medication of a preservative benzalkonium chloride. Invest. Ophthalmol. Vis. Sci..

[55] Farazifard R, Safarpour F, Sheibani V, Javan M (2005). Eye-wiping test: a sensitive animal model for acute trigeminal pain studies. Brain Res. Protoc..

[56] Pauly A (2007). New tools for the evaluation of toxic ocular surface changes in the rat. Invest. Ophthalmol. Vis. Sci..

[57] van Bijsterveld OP (1969). Diagnostic tests in the Sicca syndrome. Arch. Ophthalmol..

[58] Byun YS, Kang B, Yoo YS, Joo CK (2015). Poly(ADP-Ribose) polymerase inhibition improves corneal epithelial innervation and wound healing in diabetic rats. Invest. Ophthalmol. Vis. Sci..

[59] Gonzalez-Coto AF (2014). Expression of cholecystokinin, gastrin, and their receptors in the mouse corneacholecystokinin and gastrin in the cornea. Invest. Ophthalmol. Vis. Sci..

